# Study of Multiscale Fused Extraction of Cropland Plots in Remote Sensing Images Based on Attention Mechanism

**DOI:** 10.1155/2022/2418850

**Published:** 2022-09-05

**Authors:** Xu Song, Hongyu Zhou, Guoying Liu, Brian Sheng-Xian Teo

**Affiliations:** ^1^School of Computer and Information Engineering, Anyang Normal University, Anyang 455000, China; ^2^School of Graduate Studies, Management and Science University, Shah Alam 40100, Malaysia; ^3^Key Laboratory of Oracle Bone Inscriptions Information Processing of Ministry of Education, Anyang 455000, China; ^4^School of Software Engineering, Anyang Normal University, Anyang 455000, China

## Abstract

Cropland extraction from remote sensing images is an essential part of precise digital agriculture services. This paper proposed an SSGNet network of multiscale fused extraction of cropland based on the attention mechanism to address issues with complex cropland feature types in remote sensing images that resulted in blurred boundaries and low accuracy in plot partitioning. The proposed network contains different modules, such as spatial gradient guidance and dilated semantic fusion. It employs the image gradient attention guidance module to fully extract cropland plot features. This causes the feature to be transferred from the encoding layer to the decoding layer, creating layers full of key features within the cropland and making the extracted cropland information more accurate. In addition, this study also solves the problem caused by a large amount of spatial feature information, which losses easily during the downsampling process of continuous convolution in the coding layer. Aiming to solve this issue, we put forward a model for consensus fusion of multiscale spatial features to fuse each-layer feature of the coding layer through dilated convolution with different dilated ratios. This approach was proposed to make the segmentation results more comprehensive and complete. The lab findings showed that the Precision, Recall, MIoU, and F1 score of the multiscale fusion segmentation SSGNet network based on the attention mechanism had achieved 93.46%, 90.91%, 85.54%, and 92.73%, respectively. Its segmentation effect on cropland was better than other semantic segmentation networks and can effectively promote cropland semantic extraction.

## 1. Introduction

As an important field of land use research, cropland resources can accurately serve digital agriculture and are an essential tool for formulating national agricultural policy [1, 2, 3]. In recent years, with the rapid development of remotely sensed imaging technology and the advancement of image processing techniques, the use of satellite remote sensing images to extract cropland information has a high application value in the industry and scientific community [4, 5, 6].

According to the implementation models, the extraction of cropland information in remote sensing images can be divided into the traditional image segmentation method based on artificial features and the segmentation method based on deep learning. The manual-feature-based image segmentation method can only use limited features such as color information, texture information, and spatial structure of images for image segmentation due to the limited computational performance of the computer. This process is time-consuming and ineffective in more complex cropland segmentation, such as threshold segmentation [7, 8, 9], texture analysis [10, 11, 12, 13], edge extraction [14, 15, 16], and region-based segmentation [17].

The continuous development of modern technology in the computation field contributes to the progress of the performance of these machines and the appearance of deep learning methods [18]. These methods are widely used in the computer vision procedures such as image recognition, target detection, and image segmentation [19, 20, 21, 22]. Many scholars have used the deep learning method for cropland extraction tasks in remote sensing images and have achieved better results than the traditional image segmentation method. For instance, Li et al. [23] proposed a method of cropland segmentation and contour extraction in remote sensing images based on the Mask R-CNN of the ResNet-101-RPN backbone network. Li Sen et al. [24] constructed FD-RCF (fully dilated RCF), an edge detection model applied to remotely sensed imaging. Fan et al. [25] used a feature pyramid structure and a global context module to segment remote sensing images in UNet. Paszke et al. [26] proposed the ENet model which ensures higher accuracy and a lighter and faster network. This model is suitable for being placed in removable devices with lower power consumption, but the segmented boundary is coarser and not continuous enough. Wang et al. [27] proposed an HRNet model that could process image segmentation more spatially accurate and semantically more adequate by connecting high-resolution and low-resolution maps in parallel, advancing them simultaneously, and exchanging information continuously. Shuangpeng et al. [28] proposed the EDFANet model to replace the attention module with the convolution module by using more information aggregation and putting forward a new decoder to recover the details of the feature map. Gao et al. [29] proposed a novel MMUUNet model and a segmentation strategy in two stages of thickness to eliminate the adhesion phenomenon appearing in the cropland segmentation results. The attention mechanism imitates human brain-eye vision, which can more accurately focus on and process the most important details; it is widely used in deep learning to improve the accuracy of target extraction [30, 31]. Li et al. [32] proposed a deep channel attention module, a shallow spatial attention module, and an adaptive weight-adjusted loss function to improve the recognition segmentation of irregular targets and similar objects between and within classes in remote sensing images. Marcu et al. [33] proposed a semantic segmentation model based on global-local attention. In this model, different branches establish the boundary relationships among space, channel, and object to enhance the representation of the network and improve the recognition segmentation of architectural objects and boundaries in remote sensing images. As against the traditional classical algorithm, the deep learning method can generate simple to complex multilevel feature detectors from shallow to deep through interlayer autonomous learning and better segment the complex scenes by fully utilizing image data. However, even if the high-resolution remote sensing images are rich in details, the complex types of features, pixel mixing, shadows, and other problems within the cropland are serious, making the phenomenon of “same subject with different spectra” or “different subject with same spectra” more common, and there are still problems such as blurred boundaries and low accuracy when using deep learning for cropland segmentation. Hence, novel deep learning modules must be constructed to replenish the insufficiencies of attention mechanisms and multiscale feature fusion methods.

This paper uses submeter resolution remote sensing images as datasets for semantic segmentation of farmland. Also, the proposed model can improve the network structure of UNet [34] to address issues such as blurred boundaries and low accuracy of the plot segmentation results during extraction, which are caused by the complex cropland feature types of remote sensing images. Furthermore, this research proposes a multiscale fusion segmentation network SSGNet based on the attention mechanism. The model fully extracts the features of cropland plots with the attention guidance module of the image gradient and passes them to the decoding layer by multiplying them with the features of the coding layer at different scales, causing the key components to transfer from the encoding layer to the decoding layer. This process fills the cropland with key features and makes the extracted cropland information more accurate. In addition, to solve the easy loss of vast spatial feature information in the process of constant convolution downsampling in the coding layer, a model for consensus fusion of multiscale spatial features is proposed to fuse the features of each coding layer through the dilated convolution with different void ratios. This makes the segmentation results more complete and the segmented plots more accurate. The experimental results show that the proposed network can satisfactorily segment cropland.

## 2. Research Techniques and Methods

**Figure 1:**
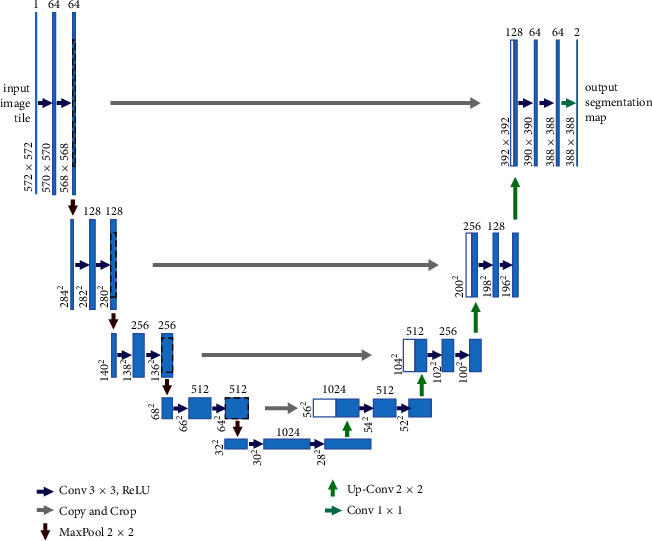
UNet network structure.

Ronneberger et al. were the first to propose the UNet network using a symmetrical encoder and decoder to make the layer-by-layer skip connection between them, by which the pixel-to-pixel relationship is obtained for precise pixel localization.  Figure 1 illustrates the network structure of UNet. It consists of two parts, the Contracting path on the left and the Expansive path on the right. The Contracting path follows the typical convolutional network architecture and comprises several repetitive structures. Each structure has two convolutional layers with 3 *∗* 3 kernel size, and these layers are followed by a modified linear unit and a max-pooling layer with 2 *∗* 2 step lengths to complete the downsampling. Each downsampling doubles the number of feature channels. At each step of the Expansive path, the deconvolution of halving the number of feature channels is used first. Then, the corresponding cropped feature maps in the Contracting path are pieced together with the deconvolution results. After each convolution, the size of the feature maps reduces, so the cropping operation is necessary. Two 3 *∗* 3 convolutions are performed on the pieced feature map, and ReLU is used as the activation function. In the last layer, the convolutional layer with a kernel of size 1 *∗* 1 is used to map the 64-channel feature map to the required number of classes. The network has 23 layers in total.

The standard UNet network architecture comprises fewer layers and a simple model. Features are extracted insufficiently in the face of multiple complex images. The most direct and effective way to increase the network layers is to upgrade the convolutional neural network's learning ability, but the pure increase contributes to too many parameters. The more complex the computation is, the more difficult the process of application is. Moreover, gradient disappearance and explosion often occur during the training process, and model optimization becomes challenging.

### 2.1. SSGNet Network Architecture

**Figure 2:**
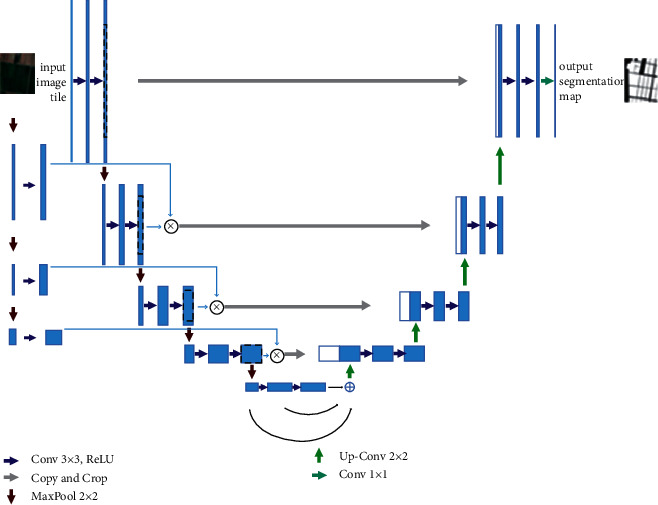
SSGNet network structure.

The proposed structure of the SSGNet network is shown in Figure 2. The network resembles the basic UNet network and adopts a coding and decoding architecture with a skip connection to fuse the high resolution of the downsampling branch with the features of the upsampling layer, improving the accuracy of segmentation and localization. The SSGNet network mainly comprises three parts: coding, decoding, and self-attention module. The self-attention module is designed between the CODEC network and the decoding-branch skip connection to calculate the correlation of positions between pixel features. This aims to strengthen the weight of valid information, fuse the image features after downsampling, and provide good basic information for upsampling.

### 2.2. Self-Attention Mechanism

**Figure 3:**
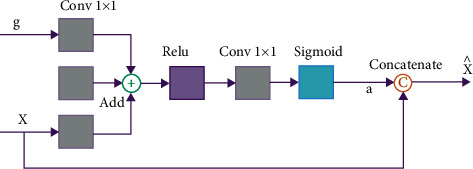
Structure chart of attention mechanism.

The core logic of the attention mechanism in computer vision [35] is “from a focus on all to focus on key points.” The structure of the attention mechanism [36] is shown in Figure 3, where *x* and *g* are the input, $\hat{x}$ is the output, and *x* is the object to be attended to. *g* is the object that provides attention to information. *x* and *g* are added element by element after a 1 × 1 convolutional transformation. The vector is then subjected to the ReLU activation function, 1 × 1 convolution, and Sigmoid activation function to obtain an attention coefficient *α*. The final output $\hat{x}$ is obtained by fusing *α* with the vector *x*. The attention coefficient *α* can identify, retain, and enhance the target region features in *x* based on the *g* input information. The formula of the attention coefficient *α* is as follows:

(1) $$\begin{array}{c} \alpha =\sigma_{2}\left(\varphi \left(\sigma_{1}\left(W_{x}x+W_{g}g+b_{g}\right)\right)+b_{\varphi }\right), \end{array}$$

where *α* ∈ [0,1], *x* and *g* are the input variables; *W*_*x*_,  *W*_*g*_, and *φ* are 1 × 1 convolution operations, playing the role of linear transformation; *b*_*g*_ and *b*_*φ*_ are the bias terms; *σ*_1_ and *σ*_2_ are ReLU activation function and Sigmoid activation function, respectively, which play the role of normalization. The output $\hat{x}$ of the attention mechanism adds the correlation weight of the *g* signal to *x*, essentially exploiting the fusion information of the two inputs. The attention mechanism performs well in modeling global dependencies as well as in computational efficiency. Therefore, the introduction of the attention mechanism enables the network to efficiently characterize the contextual relationships and enhance the representational ability of features.

The calculation of the attention mechanism is mainly divided into three steps: (1) Calculate the similarity of the attention-related query (Query) and each key (Key) to obtain the weight; (2) Use the Softmax function to normalize the obtained weight; (3) The weight and the corresponding value (Value) are weighted and summed to obtain the final attention value. In the calculation of the attention mechanism, make the Query, Key, and Value equal to get the variant self-attention mechanism of the attention mechanism. This setting can better find the relationship within the sequence, thereby making the network more efficient. Good at capturing internal correlations of data or features. The features within the same cropland plot remain the same or at least relatively the same. The plot has a slightly obvious boundary, so this characteristic is combined to propose an image gradient attention guidance module. First, the gradient of the input image is calculated, and the absolute values of gradient values in different directions are added together to find the gradient value of each point of the input image. In this chart, the gradient value of the cropland plot boundary is larger and that within the cropland is smaller, which is already a slight difference to be considered. The obtained chart is used as the gradient attention map first and then the aforementioned map as the Q (i. e., Query) in it by the self-attention mechanism. After multiplying with the coding layer's features at different scales, the result is passed to the decoding layer, causing the feature to transfer from the encoding layer to the decoding layer. This process makes the cropland full of key features and upgrades cropland extraction during the decoding process to be more accurate.

(2) $$\begin{array}{c} {\text{sSpatial}}_{X}={\text{spatial}}_{\text{conv}\left({\text{img}}_{X}\right)},\\ {\text{fFeature}}_{X}=\text{resnet}\left({\text{img}}_{X}\right),\\ {\text{rRefined}}_{X}=\;{\text{grad}}_{\text{attention}\left({\text{spatial}}_{X}\right)}⊗{\text{feature}}_{X}. \end{array}$$

### 2.3. Consensus Fusion of Multiscale Spatial Features

In the process of continuous convolutional downsampling in the coding layer, a large amount of spatial feature data is easily lost. The input of the decoding layer is mainly conditioned by the output of the lowest layer of the coding layer, so the richness of the input features of the decoding layer should be ensured during the process. Therefore, a model for consensus fusion of multiscale spatial features is proposed. This model fuses each coding layer's features by the dilated convolution with different dilated ratios. The dilated ratios of different coding layers are kept at a ratio of 2 times to ensure that the spatial features proposed by these layers are basically in the same location region. These operations help to extract the diversity features corresponding to the location area for fusion and avoid inconsistent spatial features.

(3) $$\begin{array}{c} X_{i+1}={\text{rate}}_{i}\left(X_{i}\right),\\ X^{\text{'}}={\text{rate}}_{1}\left(X_{1}\right)+{\text{rate}}_{2}\left(X_{2}\right)+{\text{rate}}_{3}\left(X_{3}\right)+{\text{rate}}_{4}\left(X_{4}\right). \end{array}$$

## 3. Experiment and Analysis

### 3.1. Experimental Data and Platform

The data used in this study were collected from within Changde, Hunan, mainly involving cropland with submeter resolution. Each cropland plot was independently labeled with two maps with the following dimensions: 13000 *∗* 12000/6000 *∗* 10000 pixels, respectively. The first one was used for training and the second one for the validation test, and cropping 768 × 768 pixels were used for training. The large image was first cropped to a small image of 768 × 768 pixels when predicted first and then pieced after predicted to conduct index calculation. The experimental computer was configured with Intel(R) Xeon(R) CPU E5-2620 v4 @2.10 GHz processor, 64 GB RAM, NVIDIA GeForce RTX 2080Ti graphics card, Python language, and PyTorch deep learning platform.

### 3.2. Evaluation Indexes

The positive and negative samples are classified into four categories: TP, TN, FP, and FN, according to the relationship between the true cases and the predicted results in the experiment. This experiment selected Recall, Precision, F1 score, and Mean Intersection over Union (MIoU) as the evaluation indexes to measure the experimental results.

Recall, also known as sensitivity, is the ratio of the number of correctly classified positive samples TP to the number of true positive samples (TP + FN), indicating the number of positive cases in the sample is correctly predicted, as shown in the following equation.

(4) $$\begin{array}{c} \text{Recall}=\frac{N_{TP}}{N_{TP}+N_{FN}}. \end{array}$$

Precision is the ratio of the number of correctly classified positive samples TP to the number of predicted positive samples (TP + FP), targeted at the prediction results. It indicates the number of positive samples in the predicted positive samples, as shown in the following equation.

(5) $$\begin{array}{c} \text{Precision}=\frac{N_{TP}}{N_{TP}+N_{FP}}. \end{array}$$

Mean Intersection over Union (MIoU) is a standard measurement for semantic segmentation. It calculates the ratio of two or more intersections and concatenations. In semantic segmentation, the two sets are both ground truth and predicted segmentations. This ratio can be morphed as the ratio of TP (intersection) to the sum of TP, FP, and FN (intersection). The MIoU is calculated on each class first and then averaged as shown in the following equation.

(6) $$\begin{array}{c} \text{MIoU}=\frac{N_{TP}}{N_{TP}+N_{FN}+N_{FP}}. \end{array}$$

The F1 score is the measurement that integrates Recall and Precision, as shown in the following equation.

(7) $$\begin{array}{c} F1\;-\text{score}=\frac{\left(1+\beta^{2}\right)\text{Precision}\times \text{Recall}}{\beta^{2}\text{Precision}+\text{Recal}}. \end{array}$$

In (7), *β* is used to adjust the weights of Recall and Precision in the F1 score. If Recall is considered important, *β* will be increased; if is considered important, *β* will be decreased; when *β* = 1, both will be considered equally important. In the cropland-image segmentation task, Recall represents how many positive cropland samples are segmented, and Precision represents how many of the segmented positive cropland samples are accurate. In cropland segmentation, we are more concerned about Recall, so we set *β* = 2 in the F1 score.

### 3.3. Model Training

Balanced binary cross-entropy and dice coefficient are jointly used as loss functions in the model training process. A mixin of functions is defined as follows:

(8) $$\begin{array}{c} \varepsilon \left(Y,P\right)=-\frac{1}{N}\overset{C}{\underset{c=1}{\sum }}\overset{N}{\underset{n=1}{\sum }}y_{n,c}\log\;p_{n,c}+\frac{2y_{n,c}p_{n,c}}{y_{n,c}^{2}p_{n,c}^{2}}, \end{array}$$

where *p*_*n*,*c*_ ∈ *P* and *y*_*n*,*c*_ ∈ Y are the target label and prediction probability of the *C* class and the *N*th pixel in the batch, respectively, *Y* and *P* are the image ground truth and prediction result, respectively, and *C* and *N* are the number of classes and pixels of the dataset in the batch, respectively, number.

**Figure 4:**
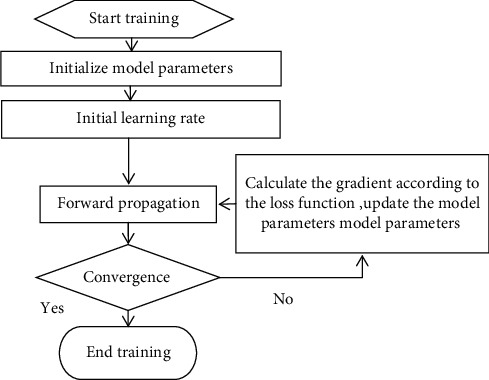
Model training process.

The Adam function is chosen as the parameter optimizer, and the initial learning rate is set to 5e-4. The number of batch training is 3, the maximum number of training iterations epoch is set to 200, and the base number of network model channels is 64. The model training process is shown in Figure 4.

### 3.4. Contrast Experiment and Analysis

**Figure 5:**
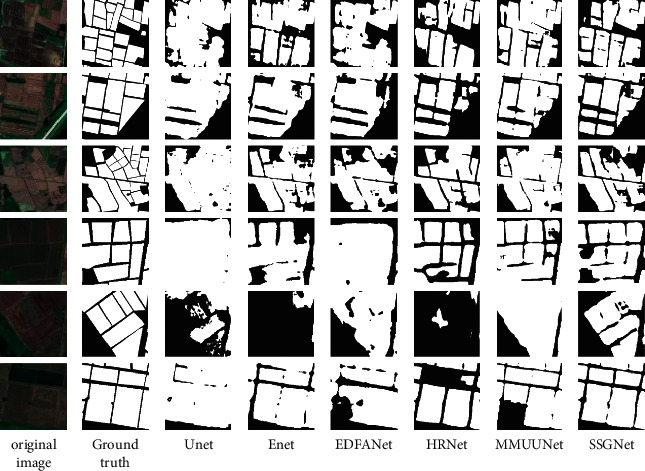
Extraction results of each network model.

To validate the effectiveness of the network model proposed in this paper, we have compared it horizontally with five semantic segmentation networks based on the dataset used to design this study. The five network models are UNet, ENet, HRNet, EDFANet, and MMUUNet, respectively, and their parameters were kept consistent with those of the original networks. The segmentation results of 6 network models were compared as shown in Figure 5. It can be seen from the figure that the interference of the geometric structure and texture features of the cropland makes the “salt and pepper effect” of the extraction results of the UNet, ENet, and EDFANet models more evident. There is an obvious misclassification and omission in cropland.

Light and shadow influence the extraction results of HRNet and MMUUNe, so there is leak detection in small-size cropland. Furthermore, the cropland plot has an incomplete boundary and rough boundary line. This paper proposes a model that should be capable of extracting detailed features of cropland features from remote sensing images. According to the characteristics of cropland plots, the local and global features can be better combined by introducing the module of image gradient attention guidance after the consensus fusion of multiscale spatial features. The geometric properties of cropland are better learned in the training process and used to obtain higher accuracy of semantic segmentation, which can elaborate boundary information. The segmented results are closest to the labels, which upgrades the edge integrity of the cropland. These outcomes prove that the leak detection and false detection issues have been initially solved.

Among the evaluation indexes in Table 1, the Precision of the network model proposed herein is 93.46%, which is 4.73%, 4.73%, 1.88%, 3.56%, 3.07%, and 3.45% higher than that of UNet, ENet, HRNet, EDFANet, and MMUUNet, respectively. The Recall is 90.91%, which is 1.06%, 0.72%, 3.28%, and 2.28% higher than that of UNet, ENet, HRNet, and EDFANet, respectively, and 0.59% lower than the highest value of MMUUNet. The F1 score is 92.73%, which is 3.47%, 1.88%, 4.06%, 3.28%, and 2.02% higher than that of UNet, ENet, HRNet, EDFANet, and MMUUNet, respectively. MIoU is 85.54%, which is 4.60%, 2.03%, 5.47%, 4.26%, and 2.28% higher than that of UNet, ENet, HRNet, EDFANet, and MMUUNet, respectively. In summary, the model proposed in this study obtained a higher accuracy when compared to other models, meeting the segmentation requirements of cropland extraction.

## 4. Conclusions

This paper puts forward an SSGNet network of multiscale fused extraction of cropland based on the attention mechanism. We introduced a novel attention mechanism model by adding a new extraction path of low-level features in the encoding layer, using the module for attention guidance of image gradient to fully extract the features of cropland plots. This arrangement causes the feature transfer from the encoding layer to the decoding layer to be full of the key features within the cropland, making the extracted cropland information more accurate. In addition, to solve the problem of a large amount of spatial feature information which losses easily during the process of continuous convolution downsampling, we presented a model for consensus fusion of multiscale spatial features. This aimed to fuse each-layer feature of the coding layer through dilated convolution with different dilated ratios to obtain rich context information and make the segmentation results more complete by expanding the sensory field and filtering background data. The model could combine the deep and shallow information of images to make its learned semantic features more accurate and the segmentation more precise and reduce phenomena such as missed extraction, wrong extraction, and incomplete extraction of cropland. The preliminary experimental results show that our model, compared to others such as UNet, ENet, HRNet, EDFANet, and MMUUNet, has the advantages of high accuracy and flat segmentation edge, and it superseded other semantic segmentation networks in the segmentation effect.

## Figures and Tables

**Table 1:** Comparison of evaluation indexes for network structure.

Experimental methods	Recall (%)	Precision (%)	F1 score (%)	MIoU (%)
UNet	89.85	88.73	89.26	80.94
ENet	90.19	91.58	90.85	83.51
EDFANet	87.63	89.90	88.67	80.07
HRNet	88.63	90.39	89.45	81.28
MMUUNet	91.50	90.01	90.71	83.26
SSGNet	**90.91**	**93.46**	**92.73**	**85.54**

The bold values indicate that the four evaluation indices of the network model proposed in this paper are higher than other models, which indicate that the network has a good segmentation effect.

## Data Availability

The data used to support the findings of this study are available from the corresponding author upon request.
